# Anti‐Eukaryotic Initiation Factor 2B‐Positive Systemic Sclerosis‐Associated Interstitial Lung Disease With Progressive Fibrosis: A Case Report

**DOI:** 10.1002/rcr2.70478

**Published:** 2026-01-20

**Authors:** Hiro Ikeda, Ryo Tachikawa, Tsuyoshi Sasada, Shuji Sumitomo

**Affiliations:** ^1^ Department of Respiratory Medicine Kobe City Medical Center General Hospital Kobe Japan; ^2^ Department of Rheumatology Kobe City Medical Center General Hospital Kobe Japan

**Keywords:** anti‐eIF2B‐positive systemic sclerosis, interstitial lung disease, progressive fibrosis

## Abstract

Anti‐eukaryotic initiation factor 2B (anti‐eIF2B) is a rare systemic sclerosis (SSc)‐related autoantibody. Longitudinal treatment data for anti‐eIF2B‐positive SSc‐associated interstitial lung disease (ILD) remain limited. We herein describe the case of a 46‐year‐old man with limited cutaneous SSc who developed lower‐lobe–predominant fibrotic ILD. Major SSc autoantibodies were negative, and an antigen‐specific panel identified anti‐eIF2B. Mycophenolate mofetil was initiated; however, serial high‐resolution computed tomography showed incremental fibrotic progression, and the diffusing capacity declined, consistent with progressive SSc‐ILD, prompting the addition of nintedanib to the ongoing therapy. This case supports testing for minor specificities when SSc‐ILD is suspected despite negative results for major autoantibodies and adds new information on the post‐treatment disease course and therapeutic selection in this serological subset. Further case accumulation with standardised longitudinal outcomes is needed to define the prognosis and treatment response.

## Graphical abstract

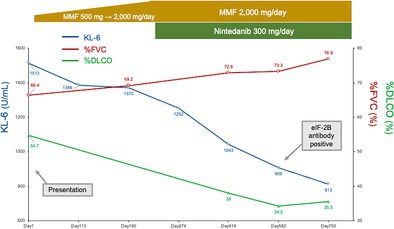

## 1 Introduction

Systemic sclerosis (SSc) is an immune‐mediated disease characterised by microvascular injury and progressive fibrosis in multiple organs, with interstitial lung disease (ILD) as a principal cause of mortality. For SSc‐ILD, contemporary guidelines recommend mycophenolate as first‐line immunosuppression; systemic glucocorticoids are minimised because of the risk of scleroderma renal crisis [1, 2, 3]. When pulmonary fibrosis is progressive despite appropriate management, guidelines support considering nintedanib in addition to background immunosuppression [1, 2, 3].

Although the autoantibody profile does not mandate a specific drug regimen [3], SSc autoantibodies help to define phenotypes and risks (including the likelihood and course of ILD), thereby informing individualised management [1, 2]. In contrast, reports on anti‐eukaryotic initiation factor 2B (eIF2B)‐positive SSc rarely detail the treatment selection, dose titration, tolerability or longitudinal outcomes [4, 5].

Herein, we report the case of a patient with anti‐eIF2B‐positive SSc‐ILD managed with stepwise mycophenolate titration and subsequent nintedanib addition, accompanied by serial biomarkers and clinical follow‐up to help delineate the therapeutic course within this rare serological phenotype.

## 2 Case Report

A 46‐year‐old man presented with exertional dyspnoea. Approximately 1 year before referral, he developed dyspnoea with fractional exhaled nitric oxide (FeNO) > 100 ppb and was diagnosed with asthma, which improved with inhaled therapy. During the preceding winter, he noted Raynaud phenomenon, chilblain‐like lesions and reflux symptoms. A screening chest radiograph showed bilateral basal interstitial opacities, and a local clinic empirically started prednisolone treatment for suspected summer‐type hypersensitivity pneumonitis. As chest computed tomography (CT) showed little improvement, the patient was referred and corticosteroids were discontinued.

At presentation, oxygen saturation was 96% (room air). History revealed Raynaud phenomenon and gastroesophageal reflux symptoms, whereas physical examination showed distal skin thickening of the metacarpophalangeal joints with fingertip pitting scars and digital ulcers, as well as abnormal nail fold capillaroscopy findings. Transthoracic echocardiography revealed preserved biventricular function without evidence of pulmonary hypertension. Autoimmune serology showed antinuclear antibody (ANA) < 1:40 and negative results for major SSc‐related autoantibodies (anti‐topoisomerase I, anticentromere, RNA polymerase III and anti‐aminoacyl‐tRNA synthetase). Anti‐SS‐A antibody was 124 U/mL (reference range: 0–9.9 U/mL), anti‐SS‐B antibody was 1.1 U/mL (0–9.9 U/mL), and anti‐cyclic citrullinated peptide (anti‐CCP) antibody was 0.6 U/mL (0–4.4 U/mL). The results of tests for myeloperoxidase and proteinase 3 anti‐neutrophil cytoplasmic antibodies were negative. The serum Krebs von den Lungen‐6 (KL‐6) was 1513 U/mL (0–499 U/mL), and surfactant protein D (SP‐D) was 509 ng/mL (0–109.9 ng/mL).

**FIGURE 1:**
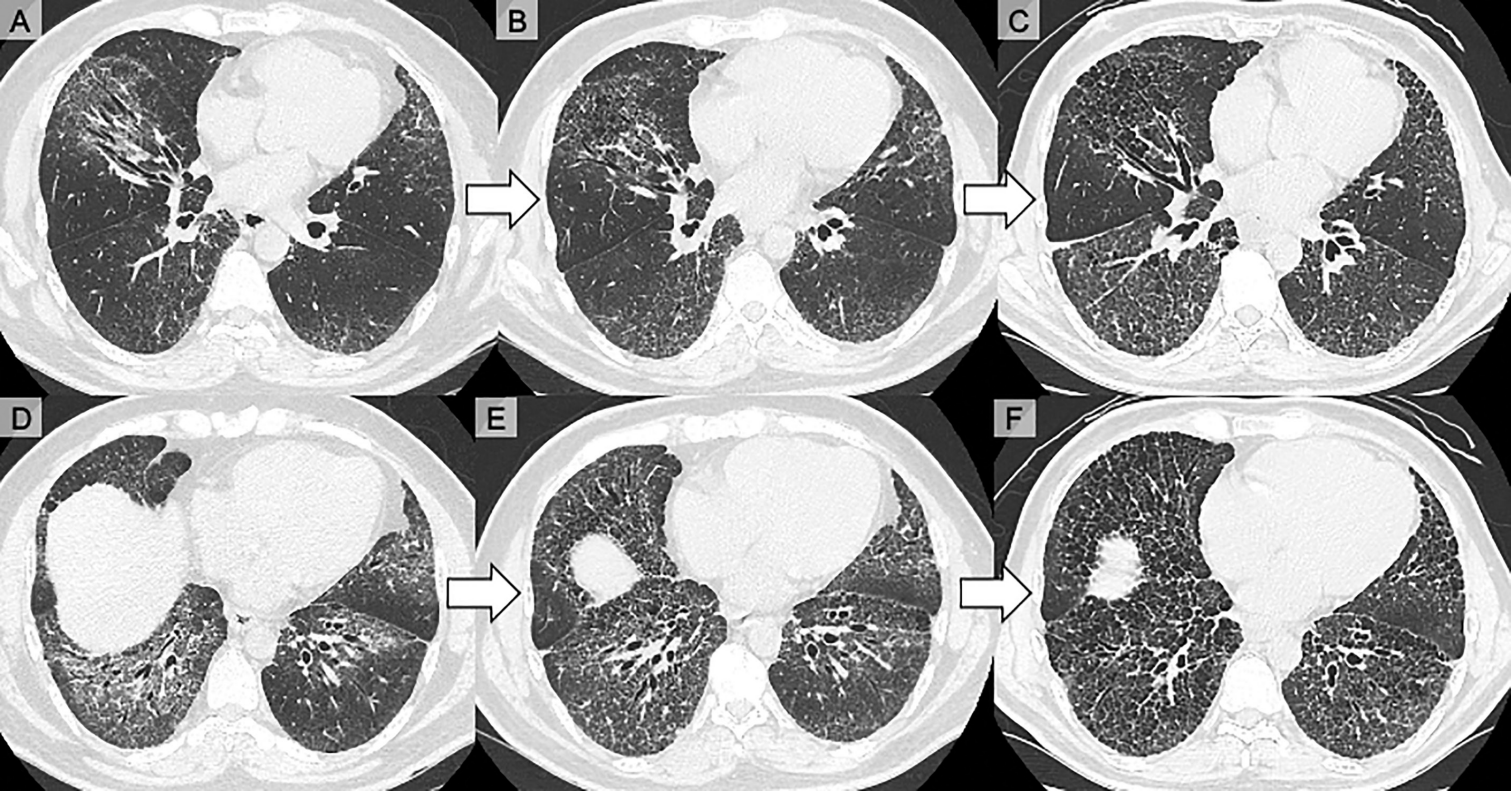
Serial chest computed tomography at two axial levels across three time points (days 1, 218 and 582). (A–C) Same axial slice on days 1 (A), 218 (B) and 582 (C). Bilateral basilar, predominantly peribronchial reticulation with traction bronchiectasis and reduced lung volume was observed on day 1; there was interval progression of fibrotic change by day 218, and further progression with increased reticulation, traction bronchiectasis and honeycombing by day 582. (D–F) Second axial slice on days 1 (D), 218 (E) and 582 (F) demonstrates the same basal‐predominant pattern with stepwise progression.

High‐resolution CT (HRCT) demonstrated lower‐lobe–predominant fibrotic interstitial changes with traction bronchiectasis involving > 20% of the lung zones (Figure 1A,D). Pulmonary function tests showed forced vital capacity (FVC) of 3.02 L (66.4% predicted), forced expiratory volume in 1 s (FEV1) of 2.32 L (59.0% predicted), FEV1/FVC of 76.82%, and diffusing capacity for carbon monoxide (DLCO) of 54.7% predicted. Bronchoalveolar lavage (BAL) revealed lymphocytes (7%), macrophages (76%) and eosinophils (15%). Based on the Raynaud phenomenon, fingertip pitting scars, sclerodactyly, abnormal nailfold capillaries, and ILD, the patient fulfilled the 2013 American College of Rheumatology/European Alliance of Associations for Rheumatology SSc classification criteria, despite negative ANA and SSc‐specific autoantibodies, and treatment was initiated. On the basis of HRCT extent (> 20%) and supported by FVC 66.4% predicted, the ILD met the criteria for ‘extensive’ disease by the HRCT–physiology staging [6, 7].

**FIGURE 2:**
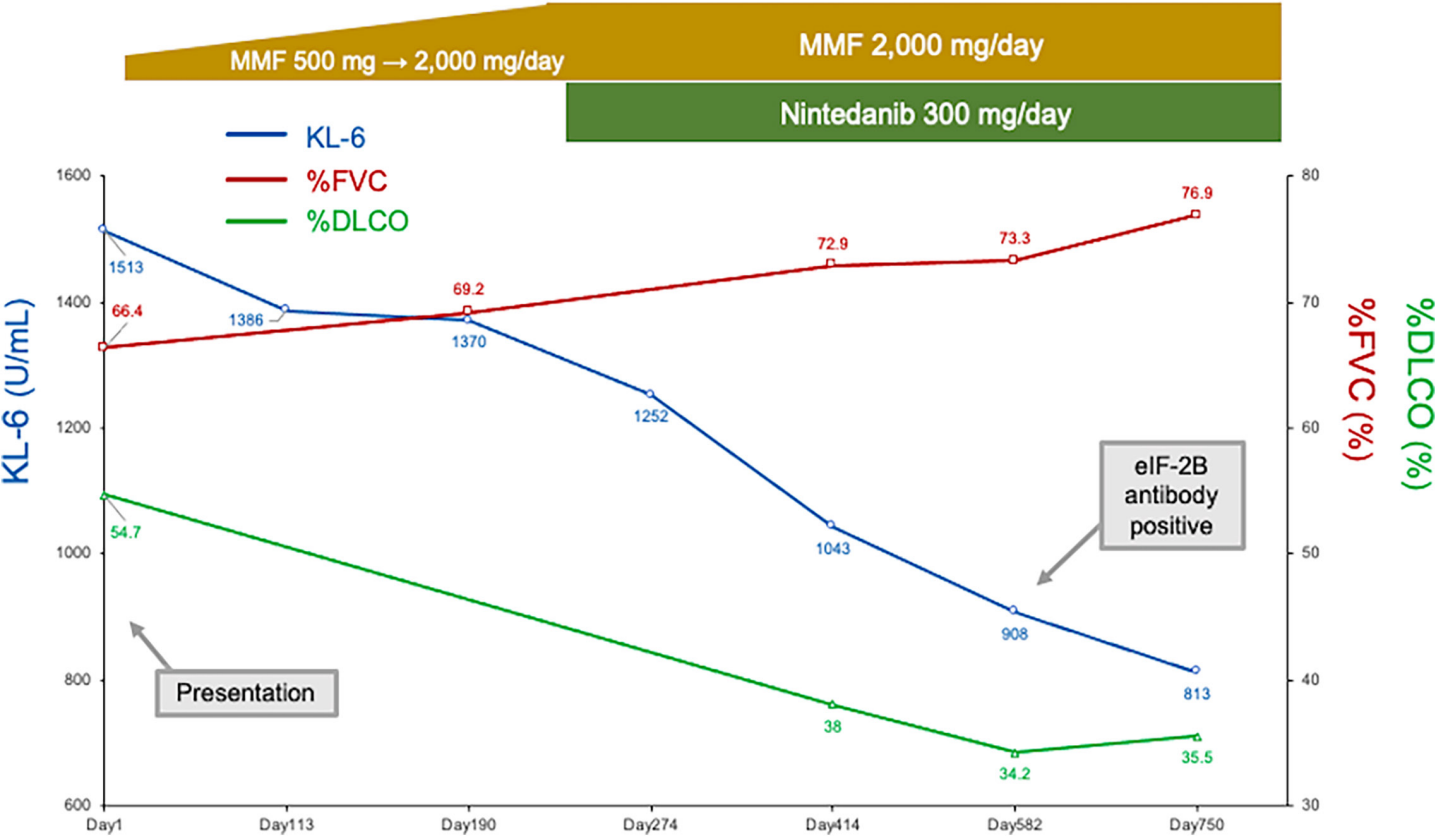
Clinical course of the patient. Serial changes in %FVC predicted, %DLCO predicted, and serum KL‐6 from day 1 (presentation) onward. Vertical markers indicate key milestones (MMF up‐titration to 2000 mg/day, addition of nintedanib 300 mg/day and confirmation of anti‐eIF2B positivity by A‐Cube). MMF, mycophenolate mofetil; FVC, forced vital capacity; DLCO, diffusing capacity for carbon monoxide; KL‐6, Krebs von den Lungen‐6; eIF2B, eukaryotic initiation factor 2B.

Mycophenolate mofetil (MMF) was initiated at 500 mg/day, but dose escalation was limited initially due to nausea, which required symptomatic management. Once nausea improved, the MMF dose was gradually increased over several months to 2000 mg/day, which represents the commonly used maximum dose in Japan (Figure 2). Because the patient exhibited limited cutaneous involvement and minimal inflammatory activity, and serial HRCT suggested a predominantly fibrotic rather than inflammation‐driven phenotype, rituximab was not prioritised. After MMF was optimised and well‐tolerated, KL‐6 and %FVC showed improving trends, whereas serial HRCT demonstrated worsening fibrotic changes and DLCO decline, consistent with progressive SSc‐ILD despite appropriate immunosuppression. Therefore, nintedanib was added as antifibrotic therapy. Bosentan was initiated for digital ischemia (pitting scars) and was titrated to 250 mg/day. Asthma remained controlled with inhaled triple therapy with a leukotriene receptor antagonist. Approximately 18 months after treatment initiation, and after MMF 2000 mg/day plus nintedanib 300 mg/day had been established, a multiplex antigen panel (A‐Cube) was submitted and returned positive results for anti‐eIF2B.

Over approximately 2 years from the start of treatment, respiratory symptoms were stable without acute exacerbations. Following nintedanib initiation, the %DLCO remained relatively stable without guideline‐level deterioration, whereas serial HRCT showed ongoing radiographic progression of fibrosis (Figure 1) [2, 3]. KL‐6 levels declined gradually, whereas FVC remained stable (Figure 2). If further disease progression occurs, lung transplantation should be considered.

## 3 Discussion

Anti‐eIF2B is a rare SSc autoantibody. When SSc is strongly suspected but major SSc‐specific autoantibodies are negative, testing for minor specificities, including anti‐eIF2B, should be considered [4, 8] In our case, serology used A‐Cube, a multiplex wet‐protein array; comparative evaluations show approximately 98%–99% agreement with enzyme‐linked immunoassay for major SSc antibodies (*κ* ≈ 0.96–0.98) and approximately 98% detection of immunoprecipitation‐validated targets, supporting analytic validity of the anti‐eIF2B result [9].

According to pooled summaries since 2016, > 90% of anti‐eIF2B‐positive patients have ILD, and diffuse cutaneous disease is frequent, with a median age of approximately 50 years and female predominance [5]. Our patient had limited cutaneous SSc, no pulmonary hypertension and no gastrointestinal symptoms, a pattern consistent with published summaries, although renal involvement has rarely been detailed [5].

As part of the diagnostic evaluation, eosinophil‐predominant BAL requires further interpretation. Published BAL data on anti‐eIF2B‐positive SSc‐ILD are very limited; in a single report with BAL differential counts, eosinophils comprised approximately 15% [5]. Our patient also had 15% eosinophils but also had coexisting asthma with markedly elevated FeNO levels, which improved with optimised inhaled therapy, suggesting an airway contribution. In unselected SSc‐ILD cohorts, BAL profiles are heterogeneous, with granulocytic patterns, especially neutrophils and sometimes eosinophils, which are commonly reported, rather than a consistent lymphocytic pattern [10]. Whether BAL eosinophilia is a recurring feature of the anti‐eIF2B subset requires further investigation.

A notable gap is the scarcity of post‐treatment time‐resolved courses for anti‐eIF2B‐positive SSc‐ILD [5]. In this case, a paradoxical increase in FVC was observed despite radiological progression of fibrosis. During immunosuppressive therapy with MMF, lower‐lobe ground‐glass opacities resolved, followed by progression of traction bronchiectasis without accompanying volume loss on CT. This constellation of findings reflects airspace enlargement due to fibrotic remodelling, with preserved or even increased lung volumes, thereby accounting for the dissociation between lung volumes and radiological progression. In contrast, DLCO declined and HRCT demonstrated worsening fibrotic changes despite background MMF therapy, meeting guideline criteria for progressive pulmonary fibrosis within SSc‐ILD and prompting addition of nintedanib in line with recommendations and trial evidence [1, 2, 3, 11]. After escalation, the %DLCO showed no further clinically meaningful decline, although radiographic progression persisted; lung transplantation is under consideration if deterioration continues. This report links explicit progression criteria to therapeutic escalation and subsequent longitudinal outcomes in this serological subset.

In conclusion, we describe an anti‐eIF2B‐positive SSc‐ILD with limited cutaneous SSc and progressive fibrosis under immunosuppression, necessitating add‐on nintedanib. When major SSc‐specific autoantibodies are negative, targeted testing for minor specificities (e.g., anti‐eIF2B) can be informative. Larger, standardised longitudinal series are needed to define the prognosis and treatment response of this serological subset.

## Author Contributions

Hiro Ikeda wrote the initial manuscript and prepared the figures. Ryo Tachikawa supervised the case and reviewed the manuscript. All the authors contributed to the writing, review and final approval of the manuscript.

## Funding

The authors have nothing to report.

## Consent

Written informed consent for publication of this manuscript and accompanying images was obtained from the patient. The authors attest that the form used to obtain consent complies with the Journal requirements as outlined in the author guidelines.

## Conflicts of Interest

The authors declare no conflicts of interest.

## Data Availability

Data sharing not applicable to this article as no datasets were generated or analysed during the current study.
